# Early life stress, literacy and dyslexia: an evolutionary perspective

**DOI:** 10.1007/s00429-024-02766-8

**Published:** 2024-03-04

**Authors:** John R. Kershner

**Affiliations:** Department of Applied Psychology and Human Resources, University of Toronto, Toronto, ON M5S 1A1 Canada

**Keywords:** Evolution, Stress, Reading, Dyslexia, CAN, VWFA

## Abstract

Stress and learning co-evolved in parallel, with their interdependence critical to the survival of the species. Even today, the regulation of moderate levels of stress by the central autonomic network (CAN), especially during pre- and post-natal periods, facilitates biological adaptability and is an essential precursor for the cognitive requisites of learning to read. Reading is a remarkable evolutionary achievement of the human brain, mysteriously unusual, because it is not pre-wired with a genetic address to facilitate its acquisition. There is no gene for reading. The review suggests that reading co-opts a brain circuit centered in the left hemisphere ventral occipital cortex that evolved as a domain-general visual processor. Its adoption by reading depends on the CAN’s coordination of the learning and emotional requirements of learning to read at the metabolic, cellular, synaptic, and network levels. By stabilizing a child’s self-control and modulating the attention network’s inhibitory controls over the reading circuit, the CAN plays a key role in school readiness and learning to read. In addition, the review revealed two beneficial CAN evolutionary adjustments to early-life stress “overloads” that come with incidental costs of school under-performance and dyslexia. A short-term adaptation involving methylation of the FKBP5 and NR3C1 genes is a liability for academic achievement in primary school. The adaptation leading to dyslexia induces alterations in BDNF trafficking, promoting long-term adaptive fitness by protecting against excessive glucocorticoid toxicity but risks reading difficulties by disruptive signaling from the CAN to the attention networks and the reading circuit.

## Introduction

Corticolimbic responsivity to environmental stressors is a variable, conditional life-force evolutionarily conserved to ensure survival of the species (e.g., Lupien et al. 2009). The merging of evolutionary developmental biology with cognitive neuroscience has accelerated our understanding of the beneficial and deleterious effects of such physiological and psychological stress response variability on emotional well-being and on attention, learning and memory (Arango-Lievano and Jeanneteau 2016; Chen and Baram 2016; Ellis and Del Giudice 2019; Kershner 2021a, b; Teicher et al. 2016; Tsigos et al. 2020).

Key corticolimbic stress system components are the hypothalamic–pituitary–adrenal (HPA) axis (McGowen and Matthews 2018) and the Locus coeruleus-norepinephrine (LC/NE) system (Bari et al. 2019). Stress responses are mediated by the release of glucocorticoids (steroid hormone cortisol in humans) by the HPA, which cross the choroid plexus blood–brain barrier, and catecholamines (mainly norepinephrine) by Locus coeruleus neurotransmission. The HPA and LC/NE are integrated, via reciprocal reverberatory interconnections, constituting a dual complimentary central automatic network (CAN) (Agorastos et al. 2019). The CAN’s primary evolutionary role, beginning prenatally, is to flexibly promote pragmatic accommodations to stressful events by maintaining and reestablishing neuroendocrine brain-wide homeostasis and individually set levels of allostasis. In contrast to homeostasis, allostasis has a wider response range and greater environmental sensitivity and refers more specifically to the ability to auto-regulate the stress hormones underlying socio-emotional and neurocognitive development. If achieved as children begin formal education, such self-control in the early grades modulates attentional arousal and cultivates a sense of personal comfort, working easily with others, and learning at a high-level of proficiency.

Under top-down surveillance by cognitive control networks, pathways interior to the CAN coalesce with changes in the environment to encourage an allostatic favorable range of adaptive functioning and resilience in response to acute and chronic stress challenges (e.g., Huzard et al. 2021). Table 1 summarizes the brain’s regulatory networks that augment allostasis via monitoring of the CAN. In a top-down hierarchy, the cognitive control network (CCN) receives and coordinates multisensory, interoceptive, and volitional inputs. The CCN is also a major outflow hub, and first to respond to goal-directed expectations and changes in afferent temperament by modulating the CAN’s range of activations. Maintaining allostasis serves to stabilize optimal internal conditions for learning and healthy physical and emotional development. From an evolutionary perspective, positive adaptation assumes increasing biological fitness, implying favorable potential for reproductive capabilities and advantages in natural selection.

**Table 1:** Controlling brain networks

Networks	Major hubs
Salience network (SN)	Right frontal insular cortex (FIC) and right frontal cingulate cortex (FCC)
Frontoparietal network (FPN)	Right dorsolateral prefrontal cortex (DLPFC) and right posterior parietal cortex (PPC)
Ventral attention network (VAN)	Right ventrolateral prefrontal cortex (VLPFC) and right temporoparietal junction (TPJ)
Dorsal attention network (DAN)	Right frontal eye field (FEF) and bilateral intra-parietal sulci (IPS)

The SN and FPN work together as a supramodal (CCN) cognitive control network (Menon and Uddin 2010; Spagna et al., 2018; Wu et al. 2020)

However, depending upon the severity, duration, and type of stress, especially prenatally and during infancy and early childhood (0–5 years), exposure to stress can exceed an individual’s normative range of resilience and stress tolerance. CAN’s internal controls modulating corticolimbic allostasis are compromised. This leads to an “allostatic overload”, whereby an excessive release of cortisol by the HPA and norepinephrine by the LC/NE may cause long-lasting adverse consequences for children’s neurophysiological and emotional development (e.g., Miguel et al. 2019; Peters et al. 2017).

Sensitive brain networks of the emotional circuitry are susceptible to early programming effects: a form of contextual learning which can induce permanent structural and functional alterations. Prenatal, perinatal, and post-natal stress can prime the developing brain for dysfunction in early childhood and pathological conditions later in life. Programming effects, such as stressors and traumatic events during this vulnerable developmental period cover a range of hazards including pre-natal maternal stress; family turmoil and violence; parental abuse and neglect; poor quality of parental care; divorce and separation, death, or incarceration of a parent; and physical/sexual abuse (Chen and Baram 2016; Kao et al. 2019). More specifically, the harmful effects of unrestrained CAN hyperactivation resulting from early-life stress (ELS) can have devastating proximal effects on fundamental neurobiological plasticity mechanisms (Salmina et al. 2021). Additionally, there are dire consequences that extend to high-risk for a wide spectrum of childhood, youth, and adult dysfunction and disease. For example, the synaptic and structural plasticity required for attention and learning (e.g., the acquisition of reading skills) and maintaining newly acquired knowledge in memory, may be impaired (Arango-Lievano et al. 2019; Halldorsdottir et al. 2019; Huzard et al. 2021; Pervanidou et al. 2020). In addition, almost every major health problem and psychiatric illness has been linked to stress. This includes depression, anxiety, PTSD, schizophrenia, multiple sclerosis, drug addiction, obesity, insulin resistance, atherogenesis, cardio and cerebrovascular events, immune disorders, and inflammatory bowel disease (Hajjo et al. 2023; Teicher et al. 2016; Tsigos et al. 2020).

Fortunately, two strategic pathways have evolved to counter these stress-linked threats to a child’s learning potential and safe and healthy childhood. Each produces phenotypic change resulting from the normal maturational variability of an ongoing evolutionary process (Wallace and Polien 2024). To lessen allostatic damage, such adaptations should engage autonomously when stress exceeds an individual’s level of tolerance, guided by age, genetic makeup, and environmental history. Both are adaptive phenotypic re-organizations of the CAN: re-sets orchestrated by natural selection and designed to mitigate or at least partially offset the negative effects of such dysregulated and heightened CAN mobilization (Ellis and Del Giudice 2019; Iwata et al. 2023; Peters et al. 2017). The most frequent adaptation, termed “vigilant”, adjusts to stress by coping with an up-regulated CAN and the second, “habituation”, down-regulates the CAN by buffering the HPA axis. Of the components of the CAN, the HPA axis, interacting with the prefrontal cortex (PFC) and hippocampus, tracks the environment and triggers regulation of the two strategies (Ellis and Del Giudice 2019). Both options are evolutionary wagers. Motivated by survival and reproductive advantage, they seek a match of the CAN’s internal state of cellular activity to the current and predictive nature of the external assault of high stress environments (see Petrullo et al. 2023 for current thoughts on evolutionary match-mismatch theory). Notably, as in most evolutionary adaptations, there are trade-offs between costs and benefits. The vigilant strategy aggressively manages ELS promoting early adaptability by elevated anxiety and greater social skills, accelerated neuronal maturation, and earlier reproduction. But prolonged CAN hyperactivation leads to long-term toxic effects on neurological, mental, and physical health. Alternatively, habituation by dampening CAN responsivity protects against long-term adverse effects on adaptability, but may be associated with low emotionality, slower development, and later reproduction. (See Ellis and Del Giudice 2019 for a detailed description of these two life-strategy adaptations).

The field is enriched with studies of ELS focused on the effects of allostatic overload on disease and psychopathology in adults. Absent from the extant literature base is research addressing the effects of ELS, mediated by the CAN, on children’s learning ability. This is unfortunate in view of: (1) basic research demonstrating the formative role of stress in modulating the underlying cellular and molecular neurophysiology of learning (e.g., Arango-Lievano et al. 2019); (2) large-sample demographic evidence showing a dose–response relationship in early school grades between the incidence of ELS and academic performance (Turney 2020); and (3) theoretical arguments proposing that exposure to ELS may be a causal factor in children’s learning disabilities and dyslexia (e.g., Burenkova et al., 2021; Theodoridou et al. 2021).

**Fig. 1:**
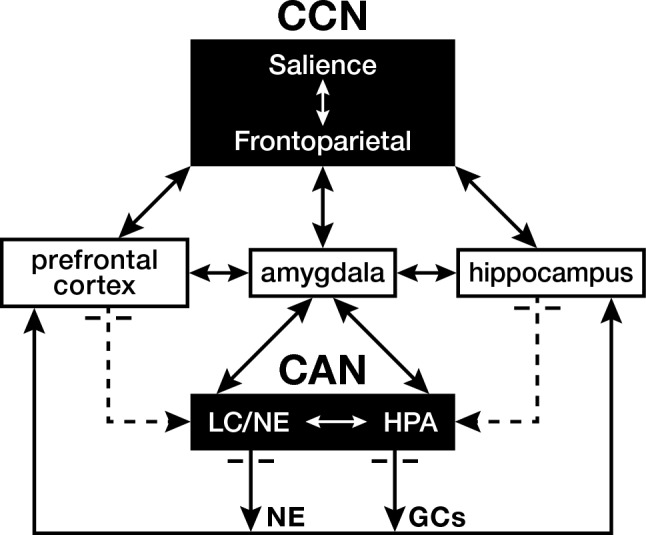
Diagram of the interactional pathways of the Corticolimbic stress system showing top-down control by the Cognitive Control Network (CCN) and bottom-up regulation by the Central Automatic Network (CAN). The CCN responds to emotional circumstances and goal-directed incentives by alerting the amygdala, which initiates complimentary activation of the hypothalamic–pituitary–adrenal axis (HPA) and the Locus coeruleus-norepinephrine system (LC/NE). The CAN’s production of glucocorticoids (GCs) and norepinephrine (NE) is modulated by feedforward and negative feedback loops with the prefrontal cortex and hippocampus to maintain allostasis of the stress system while under continuing surveillance by the CCN

The current review builds on previous reviews and theoretical analyses of ELS and reading disability or dyslexia (Kershner 2019a, b; Kershner 2020; Kershner 2021a, b). In brief, one feature of these papers conceptualized dyslexia as the cost of a beneficial evolutionary adaptation to marginal stress levels or in individuals with a low bar for stress tolerance. But left unexplored were (1) the nature of such an adaptation in view of the alternative vigilant and habituation strategies, and (2) the significance of interactions between the HPA axis and the LC/NE system. This paper addresses these outstanding issues in an endeavor to refine an evolutionary model of dyslexia. Figure 1 is a parsimonious diagram of the Corticolimbic stress system depicting CAN’s main interconnectivity pathways, and top-down regulation by the Cognitive Control Network (CCN) involved in maintaining allostasis during stress.

The first section discusses the phylogenetic origins of stress and co-evolutionary interdependence of the brain’s accommodations to stress with the evolution of the brain structures involved in learning. This covariation between stress and learning carries revealing implications for early child development. The CAN has evolved to adaptively modulate accelerated brain features engaged in both learning and emotional stability. The second section is an overview of the CAN’s normative neurobiological workings under controlled levels of typical everyday tensions and anxiety. In the third section, the Ventral (VAN) and Dorsal (DAN) attention networks, guided by the CCN and fueled by the CAN are shown to have a major role in the multimodal processing of sensory stimuli and as top-down controllers of the visual form area (VWFA) in the grapheme to phoneme correspondence essential to reading. The fourth section describes the theoretical influence of CAN’s breakdown under stress on children’s capacity in grade-school to acquire beginning reading skills. This section includes a speculative proposal suggesting that individuals diagnosed as dyslexic may be distinguished from the general academic underachiever by their stress-overload adaptation strategies. The hypothesis predicts that each may produce a different profile of interactions between the HPA and LC/NE systems. Both are positive adaptations to ELS, but each comes with a liability to children’s learning potential. Vigilance is likely to be a response to chronic and severe ELS and result in a broad variety of cognitive deficits; while habituation is associated with milder stress exposure which becomes a causal factor in developmental dyslexia. And of equal importance, irrespective of such strategies, by impairing beginning reading skills ELS presents a barrier to the proliferation of literacy in society. The final section concludes with a discussion. The focus of the paper is on the CAN’S role in beginning reading and its allostatic breakdown as a potential risk factor in dyslexia.

## Evolutionary origins

An unresolved debate in evolution is why the human-specific brain features that produce advanced cognitive abilities also make us susceptible to neuropsychiatric disorders (Mangan et al. 2022). A commonsense idea linking accelerated brain changes in hominid evolution to (1) emerging cognitive abilities and (2) adverse outcomes is that they are in a high state of entropic neuroplasticity. Such openness to environmental perturbations provides weaker damage protection and greater susceptibility to disease (e.g., Pattabiraman et al. 2020). This concept has empirical support. For example, the expansion and reorganization of the prefrontal cortex (PFC) underlying complex cognitive processing and its derangement in schizophrenia and numerous neurological disorders is well established (Woo et al. 2021). However, missing from the discussion has been the possibility of a unifying mechanism: an evolutionary adaptation that can accommodate the broad range of brain features that may either promote or impair cognitive and emotional development.

There is mounting sentiment that the CAN’s management of responsivity to stress, comprised of the HPA axis and the LC/NE system, meets the criteria for such a double-edged centralized coordinating mechanism. CAN’s diversity of actions at the brain’s cellular, synaptic and network levels are spatially and temporally distributed to influence common regions involved in regulating emotions and the basic learning computations underlying higher cognition. CAN’s production of glucocorticoids and norepinephrine is modulated by feedforward and negative feedback loops to the amygdala, PFC, and hippocampus, three key intercommunicating regions controlling emotions, memory, and learning (Agorastos et al. 2019; Kershner 2020; Pervanidou et al. 2020; Tsigos et al. 2020) (see Fig. 1). Experimental evidence has shown that the CAN triggers a cellular non-linear, inverted U-shaped stress–response curve, with ancient evolutionary origins (Schirrmacher 2021). Such a biphasic response transmission by the CAN over these signaling pathways conforms to its double-edged character. Outcomes vary dramatically. Lower stress levels promote vitality, emotional health and learning potential, while stress overloads pose a risk for emotional disturbance and cognitive impairment (Fisterwald and Alberini 2014; Lupien et al. 2009).

In other words, CAN’s modulating influence has a bidirectional neurophysiological and behavioral effect on human-specific features participating in stress and learning. There can be little doubt that responsivity to stress has been interdependently coupled with and supported by learning throughout evolutionary history (Schirrmacher 2021) and is interwoven dynamically during child development (Fisterwald and Alberini 2014). Even single-cell organisms cannot survive without the inheritance of epigenetic memories to inform their biphasic responsivity to physical stressors. This is an evolutionary principle that generalizes to all life forms (e.g., Schirrmacher 2021). In humans, adaptive behavioral outcomes to stress involve access to programmed knowledge, oftentimes acquired covertly through past exposure to stress, and the ability to assimilate and memorize the circumstances surrounding stressful events. With certainty, coping with stress successfully requires learning as much as possible about the stressor. And the acquisition and retention of such knowledge depends on stimulation from the stress systems. Thus, the CAN is an evolutionary mainstay that has evolved to guide the co-evolutionary interdependence of neuronal regions responsible for stress management and learning ability.

## Can circuitry

For a better understanding of CAN’s adaptive role in learning, it would be instructive to review the inner mechanics and interaction of the CAN’s two stress systems.

### HPA axis

In response to stress, the CCN top-down informed amygdala initiates an endocrine cascade by: (1) activating the hypothalamic paraventricular nucleus (PVN) to release corticotropic-releasing hormone (CRH); which (2) stimulates the anterior pituitary to release adrenocorticotropic hormone (ACTH); which (3) causes the adrenal cortex to release cortisol as the HPA’s final product. Cortisol transits the blood–brain barrier and combined with BDNF activity-dependent signaling, acts as a glucocorticoid (GC) ligand in binding with mineralocorticoid (MR) and glucocorticoid (GR) receptor-phosphorylation sites found throughout the brain. BDNF is a brain-derived neurotropic factor, with BDNF/TrkB receptors concentrated in the amygdala, PFC, and hippocampus where it regulates GR sensitivity and neuroplastic changes affecting learning and memory in response to stress (Grigorenko et al. 2016; Miranda et al. 2019). BDNF-dependent phosphorylation stabilizes cellular metabolism and prepares the cell nucleus for epigenetic gene transcriptionally active processes. As stress exceeds basal levels, GC/GR receptor binding takes precedence over GC/MR and serves two important adaptive functions. Such binding promotes neuroplasticity in the PFC and hippocampus, which regulates the HPA’s production of corticosteroids via inhibitory return pathways to the HPA (McGowan and Matthews, 2018). Thus, the HPA via connectivity to the PFC and hippocampus, which are also rich in GR receptors, fulfills a vital function in stress management by sustaining its own negative feedback loop to regulate allostasis of the HPA (see Fig. 1).

Secondly, BDNF-dependent GC/GR receptor binding, leveraged by the production of cortisol, conveys contextual information about the environment to the gene transcriptional processes essential to learning and memory (Arango-Lievano et al. 2019; Huzard et al. 2021). Upon GC activation, the newly energized receptors pass from the cytosol to the cell nucleus, where they transactivate or depress the mRNA expression of specific genes involved in the protein consolidation of new dendritic spines, spine maintenance, and synaptic plasticity. Attempting to synchronize the external pressure of stress with the internal state of the amygdala, PFC, and hippocampus, GR receptor activation engages all three in a cognitive supporting framework for their parallel roles in stress control. Together, they respond to the environment through implicit spatial and associative memories and the regulation of executive behaviors (e.g., Kalik and Rakic 2022; Liu et al. 2023; Simac et al., 2021). Therefore, the HPA’s control of GC production has a formative impact on children’s learning. However, the full scope of the CAN’s role in learning requires input from the LC/NE system.

### LC/NE system

The LC/NE system originates in the Locus coeruleus (LC), a small nucleus in the brainstem. Under CCN supervision and responding to convergent sensory information and goal-directed intentions, the amygdala alerts the LC to release norepinephrine (NE). NE serves multiple purposes. First, NE released to the PFC and hippocampus activates a self-regulatory negative feedback loop to maintain allostasis of the LC/NE system (Morris et al. 2020; Tsigos et al. 2020). Second, NE modulates the gain efficiency of higher order signal transmission in the brain networks controlling arousal and attention to behaviorally relevant stimuli (Totah et al. 2019). And third, the release of NE throughout the brain modulates the disbursement of GABA and glutamate, the brain’s primary inhibitory and excitatory neurotransmitters (Peters et al. 2017). As a neuromodulator system, the LC/NE is a highly evolvable master regulator of neuroplasticity, capable of maintaining a regional and network balance of excitation and inhibition by targeting specific neuronal populations (Huzard et al. 2021; Totah et al. 2019). The LC/NE system’s distributed modulation of the brain’s major neurotransmitters serves two functions complimentary to the HPA.

The LC/NE system’s response to stress is immediate. Upon activation, the LC, which is the brain’s only source of NE, releases NE to the hypothalamic paraventricular nucleus (PVN) which activates the HPA axis in a collaborative stress response. Reciprocally, the PVN discharges corticotropic releasing hormone (CRH) to coactivate the LC (e.g., Tsigos et al. 2020). Thus, the LC/NE system and the HPA axis are bound to respond in parallel to stress via bidirectional connectivity between the LC and the PVN. Balanced synchrony is achieved by each stimulating the other through CRH receptors (CRHR1) in the LC, and alpha-1 adrenergic receptors in the PVN.

The second complimentary function of the LC/NE system is obligatory to CAN’s conserved evolutionary role in affect and learning. Successful GC/GR binding requires presynaptic glutamatergic excitatory input (Arango-Lievano et al. 2019), which may be modulated by NE released from the LC (Huzard et al. 2021). Therefore, the LC/NE system’s interaction with local glutamatergic receptors (glutamate receptor A1) in the PFC and hippocampus appears to be necessary for (1) reinforcing their negative feedback control of glucocorticoid production and (2) coordinating the stress response with their cognitive functions in associative learning and the retention of new information. Indeed, synaptic plasticity, cellular phosphorylation, and the epigenetic gene transcriptional processes required in learning depend on successful BDNF-dependent GC/GR binding (Arango-Lievano and Jeanneteau 2016) which, in turn, depends on HPA allostasis and input from the LC/NE system.

In summary, stabilization of the CAN in coping with early-life stressors (ELS), composed of the pooled functions of the HPA axis and the LC/NE system, sets a high achievable mark for the full realization of a child’s learning potential. Lastly, the LC/NE system plays a more specific and prominent role in reading, which involves an understanding of the importance of the visual word form area (VWFA).

## Can and reading

### Visual word form area (VWFA)

In reading, a left hemisphere brain circuit, activated by letters and written words, transforms visual symbols into speech sounds and meaning in several hundred milliseconds. The nucleus of this circuit is a small region in the ventral occipital-temporal cortex known as the VWFA, which is unique in its dual interconnectivity to the brain’s main territories for processing language and visuospatial attention. The VWFA is thought to be organized functionally along a topological posterior to anterior sequential processing gradient. The VWFA accesses areas responsible for (1) rapid visual simultaneous processing by posterior connectivity to the left dorsal attention network’s (DAN) Intraparietal sulcus (IPS) and (2) speech production and comprehension by anterior connectivity to Broca’s area in the left inferior PFC (Brem et al. 2020; Chen et al. 2019; Caffarra et al. 2021; Vogel et al. 2014; White et al. 2023; Yablonski et al. 2023; Zhao et al. 2021; Zhao et al., 2016). Moreover, these avenues of communication with the VWFA rest on the developmental integrity of two large white matter tracts known as fascicles: (1) *connectivity between the anterior subregion of the VWFA (VWFA-2) and Broca’s area is *via the long segment of the arcuate fasciculus (AF); and (2) between the posterior VWFA (VWFA-1) and the DAN via the vertical occipital fasciculus (VOF). Broca’s area, via the AF, is an integral component of the dorsal reading route and organized topologically to work with the anterior VWFA in verbally processing letters and words (cf. Yablonski et al. 2023). The left hemisphere IPS plays an earlier and more comprehensive role providing top-down feedforward inhibitory controlling inputs to VWFA-1, and for feeding back inhibitory integrated sensory signals to VWFA-1 and VWFA-2 to strengthen the associations between phonological and orthographic information (Kay and Yeatman 2017; White et al. 2023).

Indeed, over the last decade, remarkable strides have been made in understanding the critical function of GABAergic or inhibitory interneurons as regulators of cortical information flow between the brain’s controlling networks and their principle down-stream processing regions (Fishell and Kepecs 2020; van Oostrum et al. 2023; Roux and Buzsaki 2014). All excitatory synaptic afferents to targeted dendritic domains are relayed by inhibitory interneurons. They are evolutionarily conserved, originating in the embryonic migration of GABAergic progenitor cells. In the IPS to VWFA pathway, i.e. the VOF, they function in feedforward and feedback inhibitory microcircuits which rout, filter, and modulate excitatory activation, regulating (1) the gain and timing of cell firing (2) integrating the pooled functions of the IPS and VWFA and (3) powering the phase/amplitude couplings (i.e., theta/gamma) essential for phonemic processing. In short, the association of written language with their speech sounds by the VWFA depends on the interplay of inhibitory interneurons. Thus, the VWFA with its dual interconnectivity is uniquely positioned and engendered with the controlling microcircuits to be a domain-specific computational epicenter for reading.

Such a reading circuit raises important developmental questions. First, in beginning readers, why and how does the VWFA first develop sensitivity to written language? What is the part played by genetics vs. the environment? And secondly, how do the two earlier activated regions of the circuit (i.e., IPS and VWFA-1) interact to facilitate beginning reading? Children learn to associate the unfamiliar visual characters of the alphabet with the sounds of their language and eventually learn something meaningful from the written word. No neuro-educational challenge could be greater.

### Development of VWFA’s sensitivity to print

To answer the first question, we need to acknowledge the absence of evidence of biological selection pressures for reading (e.g., Taylor and Vestergaard 2022). The cultural expectation for children to read is too recent in history for evolution to have carved out an adaptive genetic basis specific to reading. In addition, only about half of the children at familial risk become dyslexic, and after controlling for environmental adversity, absence of home literacy opportunities, and health issues, genetic risk loses predictive significance (Dilnot et al. 2016). Thus, to be able to read or not has no advantage or disadvantage in biological adaptability. Simply, there is no gene or intrinsic prewiring for reading or for the VWFA’s sensitivity to print.

Rather, the VWFA acquires sensitivity to print through experience. The VWFA is not responsive to written language until reading is introduced in the early grades. Neuroimaging studies have shown that print sensitivity emerges along with development of the extended reading circuit only as children acquire letter–sound associations and can read single words at a fast pace (Frago-Gonzalez et al. 2021; Moulton et al. 2019). During beginning reading instruction, the VWFA, VOF, and IPS, developed in parallel, reaching peak-levels of activation which gradually moderated over the year. White matter imaging showed stronger connectivity during the year of instruction between the VWFA and its VOF cortical tract terminations in the IPS. This suggests that an ancient and established evolutionary circuit, i.e., the VWFA, VOF and ISP, has been co-opted to become a central component of the brain’s reading center in response to the evolutionarily more recent and novel processing demands of decoding written language. Indeed, post-mortem and diffusion tractography studies suggest that this circuit evolved and continues to function as a synergistic system integrating visual information between the dorsal and ventral visual systems (Jitsuishi et al. 2020; Kay and Yeatman 2017; Vogel et al. 2014). In addition, although the VWFA is only weakly connected to the IPS prior to the need to decode print, the IPS, in collaboration with the right frontal insular cortex (rFIC), is one of the brain’s top-down centers for modality independent multisensory processing (Anderson et al. 2010; Chen et al. 2015; Porada et al. 2021; Rohe and Noppeney 2018). The IPS is enriched with multisensory neurons making it a primary site for the general-purpose capacity of integrating ecologically valid multisensory stimuli from separate channels into composite mental representations: an evolutionarily adaptive function. This scenario invites the hypothesis that a major reason for reading to favor co-option of the VWFA-VOF-IPS pathway is the IPS’s integrative multisensory function: essentially a pre-wired potential serving as a template for the more specific VWFA’s print-to-sound integrative processing required in learning to read. The brain may have taken a path of least resistance. However, this open issue turns out, for an understanding of the regional interactions of the reading circuit, we need to examine their specific role in letter-sound associations.

### VWFA reading circuit and letter-sound mapping

Notably, slow, or inaccurate letter-to-sound mapping is the core behavioral defining characteristic of dyslexia according to widely held definitions (Poulsen et al. 2023). There can be little doubt that prior to semantic interpretation, the ability to consolidate the conversion of graphemes to phonemes by the VWFA is fundamental to the reading process. However, core processors are not organized adaptively to act in isolation.

Information processing centers in the brain require top-down controlling inhibitory inputs from regions and networks outside of the central processing module (Petersen and Posner 2012). Their computational engagement depends on inhibitory modulation of cortical function driven by activation of GABAergic interneurons (Huzard et al. 2021). Of the two interconnecting regions of the reading circuit capable of cognitive control of the VWFA, fMRI brain imaging research suggests a preferential contribution to the consolidation of letter–sound conversion by DAN’s bilateral IPS and a minor role for frontal regions (Kolodny et al. 2017)*.* Posterior regions of the VWFA, i.e., VWFA-1, are activated by orthographic features of words approximately 200 ms earlier than the delayed processing of higher order language features in the anterior VWFA-2 region (Caffarra et al. 2021).

The LC/NE system influences this conversion process indirectly by modulating the attention network’s inhibitory controls over the VWFA reading circuit. The LC projects NE, via the right thalamus, to the attention networks where the DAN’s left IPS is structurally and functionally organized for top-down controlling interactions with the VWFA (Aston–Jones and Cohen 2005; Corbetta et al. 2008; Giller et al. 2020; Morris et al. 2020; Petersen and Posner 2012). However, a comprehensive understanding of all the players in the conversion process requires a more detailed review.

### CCN, LC/NE system, attention networks and VWFA

**Fig. 2:**
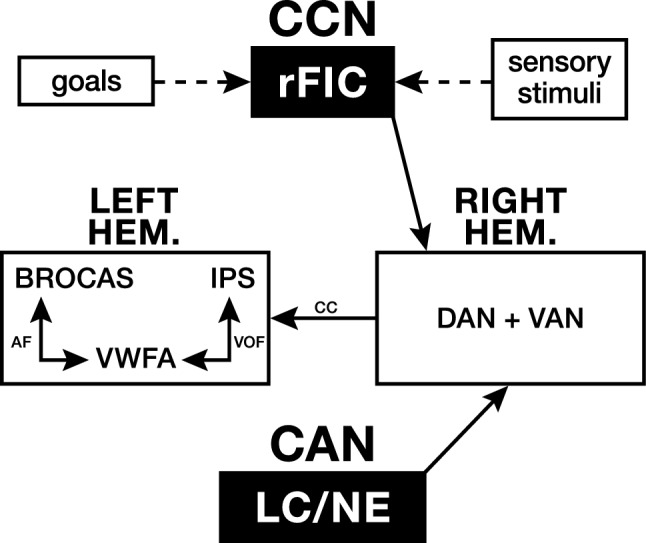
Diagrammatic model of the proposed management hierarchy of the extended reading circuit. When learning to read: (1) in the right hemisphere, the Dorsal attention network (DAN) is activated top-down by the right frontal insular cortex (rFIC) of the Cognitive Control Network (CCN), while the Ventral attention network (VAN) is activated bottom-up by the Locus coeruleus-norepinephrine system (LC/NE); (2) signaling from both modulates the collaborative engagement of the attention networks interhemispheric inhibitory controls (by corpus callosum or CC projections) over the left hemisphere reading circuit; where (3) DAN’s intra-parietal sulcus (IPS) has a leading controlling function, via the ventral occipital fasciculus (VOF), over print-sound mapping by the posterior visual word form area (VWFA-1); and (4) reading comprehension relies on VWFA-2 anterior connectivity via the arcuate fasciculus (AF) with Broca’s area

VAN is the bottom-up gateway to the attention networks. The VAN is strongly right hemisphere lateralized and interconnected with the DAN by: (1) the second branch of the superior longitudinal fasciculus (Chica et al. 2018; Thiebaut de Schotten et al. 2011); (2) the right posterior middle frontal gyrus (Corbetta et al. 2008); and (3) the right inferior frontal junction (Corbetta and Shulman 2011). VAN’s bottom-up activations, modulated by LC/NE signaling, support DAN’s posterior top-down interhemispheric allocation of attentional resources. Together, they coactivate to control exogenous and endogenous attentional processing (Zhao et al. 2021). According to Corbetta et al., (2008), the LC/NE system fuels flexible tonic/phasic modes of processing, which subserve the orient/interrupt/reorient functions of the VAN and DAN. The LC/NE system projects to the VAN which: (1) gates distractions by down-regulating tonic activity, enhancing DAN’s phasic activity and exogenous orientation to focus on an attended task (e.g., reading); (2) combines with the DAN in disengaging and reorienting attentional focus (e.g., the sweep of visual fixations in reading); and (3) regulates DAN’s left hemisphere endogenous attentional control functions (e.g., interconnectivity between the left IPS and the VWFA for print-sound integration). Thus, the LC release of NE interacting with local glutamate may modulate all three functions in reading. (See Fig. 2 for the interhemispheric components of the extended VWFA reading circuit).

To summarize, reading depends on the CAN’s stress-regulated production of glucocorticoids and LC release of NE for successful BDNF-dependent GC/GR receptor binding. In effect, children cannot easily learn the alphabetic principle in reading without stress-managed allostasis of the HPA and measured release of NE from the LC/NE system to fuel tonic/phasic modes of down-stream controlled processing by the attention networks. We assume that most children have adjusted successfully to the environmental stressors encountered in their life histories and are entering early grades having maintained an adaptable level of corticolimbic allostasis. They have achieved self-regulation over attention and the socio-emotional maturity important for learning readiness. When reading is introduced, an evolutionarily conserved brain circuit, centered on the VWFA, is poised for recruitment for the print-sound association demands of reading because of its innate but relatively undeveloped connectivity to the left IPS with its capacity for multisensory integration. As reading instruction begins, the initial stages of learning to read are supported by (1) bottom-up LC/NE modulated signaling to the right hemisphere attention networks and (2) top-down management by the rFIC and the DAN in the inhibitory feedforward and feedback-controlled interconnectivity with the VWFA where print-sound associations are consolidated for reading comprehension. For children with a well-managed CAN, although learning to read requires greater effort and dependence on attentional controls compared to fluent reading, it is not a particularly challenging process and reading can be a recreational pleasure and route to academic success.

On the other hand, it has been hypothesized that exposure to early-life stress (ELS) resulting in dysregulation of the CAN may be a significant risk factor in reading disability and dyslexia. This is an outstanding question addressed in the next section.

## Effects of ELS on reading disability and dyslexia

Two background points require emphasis. First, central features of the brain’s VWFA reading circuit have evolved since the divergence of the human lineage from bonobos and chimpanzees. Human-specific accelerated regions include (1) the right hemisphere attention networks and their frontal-parietal connectivity via the superior longitudinal fasciculus and (2) the left hemisphere arcuate fasciculus connecting Broca’s frontal area with temporal zones (Ardesch et al. 2019; Martini et al. 2013; Patel et al. 2015). Their relatively recent evolution suggests a flexible neuronal substrate ripe for co-option by the emotional and cognitive requirements of reading, but also lessens the damage protection of more established stress coping features (Pryluk et al. 2019). Thus, the ancient evolutionary origins of stress system adaptations, i.e., habituation, may offer some protection for general cognitive functions, health outcomes and longevity. But their ancient evolution predates the newly emerged reading circuit, leaving literacy and the circuit open to allostatic dysregulation. Clearly, there is a substantial evolutionary argument for an ELS/dyslexia relationship. And secondly, despite this and the strength of the theoretical association between the CAN and the neurophysiology of reading, the putative linkage between ELS and dyslexia is understudied and far from being established. Hundreds of studies have documented lifetime emotional and cognitive impairments resulting from Intrauterine and post-natal stress (e.g., Miguel et al. 2019). However, research on the specific linkage between ELS and beginning reading is lacking.

Longitudinal studies would be informative. They should begin in utero with observational data of specific stressors, combined with behavioral and biomarkers of diurnal rhythms and stress regulation, and early reading skills as the dependent measures. A lack of pertinent research leaves the issue unresolved. Moreover, studies with some relevance are incomplete in providing the information needed. For example, ELS has been related to learning disability, and failure to meet K-6 grade-level achievement in math, reading, and writing, but measures of the CAN were not in the research designs (Blodgett and Lanigan 2018; Turney, 2020). Salivary cortisol and hair cortisol are used as biomarkers of acute stress reactivity and the cumulative effects of chronic ELS. Unfortunately, studies that have linked cortisol concentrations with poor academic achievement suffer from design limitations preventing the determination of cause vs effect (for a review see Burenkova et al. 2021). In addition, there are a few studies with young dyslexia samples demonstrating atypical cortisol response; but no information was provided on associations of the cortisol response with early-life stress events (Buchweitz et al., 2021; Espin et al. 2019; Huang et al. 2020). Therefore, the question cannot be answered by a review of available research.

Consequently, our approach will be to advance the theoretical grounding of the hypothesis by examining (1) implications for reading acquisition of the CAN’s breakdown under an allostatic overload and (2) evidence of stress-related adaptation strategies that may be associated with reading under-achievement or dyslexia.

### Gene-methylation effects on the can

One genetic basis of reading resides indirectly in the elaborate epigenetic machinery of the CAN which, via HPA axis gene methylation, links environmental stress to brain-wide BDNF-dependent GC/GR receptor binding (Norman and Buttenschon 2020). Such gene–environment interactions, called epigenetics, regulate gene expression in a transcriptional process without altering DNA sequence and are inherited across generations. During pregnancy, maternal in utero stress effects on the CAN are transmitted to the developing embryo. Such fetal programming is well-known for potential negative effects on the CAN’s proper stress response later in life. In response to chronic stress, methylation attaches methyl groups to nuclear DNA or around histones to form chromatin at so-called CpG sites (Chalfun et al. 2022; Tian et al. 2023). By occupying these sites next to target genes, methylation disrupts RNA transcription resulting in gene silencing, repression, or deleterious enhancement.

Of the eight HPA genes that have been identified, methylation changes of the FKBP5 and NR3C1 genes have been associated with ELS, dysregulation of the HPA, and cognitive ability (Chalfun et al. 2022;  Matosin et al., 2018). Increased methylation typically down-regulates gene transcription, while reduced methylation upregulates transcription. Both genes are key endogenous regulators of GC/GR receptor binding. The FKBP5 gene regulates the environmental sensitivity of GRs, whereas the NR3C1 gene codes for the GR receptor. It appears that both are consequential supports for the HPA negative feedback controls and cognitive functions of the PFC, amygdala, and hippocampus.

Two experiments demonstrated ELS-cognitive effects mediated by alterations in expression of these genes. Subjects whose mothers experienced an earthquake during their second trimester showed atypically high methylation of the NR3C1 gene 38 years later which correlated strongly with tests of working memory (Wang et al. 2022). The second trimester is a dynamic phase in the differentiation of thalamic glutamatergic neurons, the distribution of GABAergic neurons, and the onset of gliogenesis (Kim et al. 2023). This fetal reprogramming was thought to result from diminished NR3C1 expression, desensitizing the GC/GR controls of the HPA negative feedback loop, with long-term disruptive effects on neural structure and synaptic transmission in the PFC. A second study reported that children with two copies of a risk FKBP5 haplotype (four specific FKBP5 gene variants inherited together) who were exposed to parental/caretaker violence during the first two years of life had a developmental history of high cortisol reactivity during that time, followed by emotional and behavioral problems and poor academic achievement in grades 1, 2, and 5 (Halldorsdottir et al. 2019). The results were interpreted as high FKBP5 expression interfering with GR feedback regulation coupled with down-stream FKBP5 over-expression affecting the volume and connectivity of the PFC, amygdala, and hippocampus.

Thus, the risk of ELS dysregulation of the CAN is determined partly by the nature and timing of gene–environment interactional variability with methylation-altering gene expression profiles. Children with specific combinations of single nucleotide polymorphisms (SNPs) exposed to ELS are at risk for serious psychiatric, cognitive, and health problems. It follows logically from this that children’s receptivity to reading instruction in the early grades will depend on the CAN’s BDNF-energized GC/GR binding, which is informed by gene-environment transcriptional processes potentially dysregulated by methylation of HPA axis genes. Only individuals with specific gene expression profiles are at risk and only when exposed to ELS during critical periods of brain development.

And finally, from an evolutionary perspective such gene-methylation outcomes are generally consistent with the “vigilant” stress adaptation strategy. Excessive GC exposure in utero or early childhood may program and alter GC signaling permanently, conferring a short-term advantage but risk of long-term emotional and cognitive deficits including under-achievement in reading (e.g., Tsigos et al. 2020).

## BDNF effects on the can

BDNF signaling is a stress-reactive, neurotropic factor released by the brain to regulate the brain-wide synaptic plasticity of GC/GR binding, with BDNF/TrkB receptors concentrated in the PFC, amygdala, and hippocampus (Huzard et al. 2021; Kershner 2020). BDNF and GCs are complimentary in their behavioral roles. GC production suppresses BDNF to maintain a circumscribed response range of the HPA negative feedback loop, while BDNF regulates the effect of GCs on synaptic plasticity of the GRs.

Changes in gene-methylation degrade GR receptor binding by disrupting GC secretion or GR sensitivity. In contrast, stress-induced alterations in BDNF impact the post-synaptic neuroplasticity of GR receptors, which vary from region to region. Such variability has the net effect of disassociating the HPA from the stress response of the LC/NE system (Chattarji et al. 2015; Huzard et al. 2021; Jeanneteau et al. 2019; Tsigos et al. 2020). This adaptation to ELS involves a correlated reduction of BDNF-plasticity in the PFC and hippocampus, coupled with an atypical gain of plasticity in the amygdala and its connectivity to the LC/NE system. As a result, (1) the HPA axis becomes recalibrated to greater sensitivity, which attenuates the production of cortisol but (2) elevates the release of NE from the LC via its direct signaling pathway to the attention networks (Aston-Jones and Cohen 2005; Corbetta et al. 2008; Giller et al. 2020; Morris et al. 2020; Petersen and Posner 2012). Excessive NE release can be expected to spark overactivation of glutamatergic neurons, inducing glutamate excitotoxicity in the right-hemisphere attention networks, reducing their processing flexibility and the DAN’s top-down interhemispheric inhibitory controls, via the left hemisphere VOF, over the VWFA reading circuit (Woo et al. 2021).

Thus, ELS alterations in BDNF trafficking, by adaptively buffering the HPA axis but selectively disrupting attentional control over the reading circuit, is consistent with the “habituation” stress adaptation strategy and may become a risk factor for dyslexia. In effect, regional BDNF variance can cause an asymmetrical response of the two arms of the CAN, protectively gating the toxic flow of cortisol, but incidentally compromising children’s ability to learn print–sound associations in beginning reading.

To summarize this section, changes in gene-methylation and BDNF signaling in the CAN are two pathways for ELS to upset the allostatic balance underlying children’s ease in acquiring beginning reading skills. Both stress adaptation pathways, singularly or in combination, present a “hidden” teaching challenge in early schooling and a significant impasse to the spread of literacy in society. ELS-related HPA gene-methylation effects are compatible with the expectations of vigilant adaptation, suggesting an association with the garden variety poor reader. The theoretical narrative of atypical variations in BDNF signaling aligns well with the habituation adaptation resulting in dyslexia. Hence, validation of the two adaptations may become useful in education, leading to innovative clinical tests of HPA function to differentiate the common underachiever in reading from the more severely disabled dyslexia phenotype. However, this possibility has not been addressed directly by empirical investigations, so should be viewed as a speculative hypothesis pending future studies.

## Discussion

With origins in the earliest evolutionary times, coping behaviorally with stress has relied upon a supportive transcriptional learning response, involving the programming of epigenetic transgenerational memories. From the protection and survival of single cells to multicellular organisms, learning ability has co-evolved to the present day with the CAN’s adaptive capacity to respond to stressful events. This synergistic relationship plays prominently in modulating intrauterine development and regulating allostasis during infancy and early childhood. The HPA axis during fetal development appears to be especially sensitive to stress, perhaps reflecting its eventual post-natal role as a neural integration center for the emotional controls and learning requirements of adaptive stress-coping strategies. With continuing maturation of both arms of the CAN, sensory and environmental contextual information are routed to the amygdala, PFC, and hippocampus which (1) regulate the CAN’s stress response and (2) engage in causal associative learning mediated by GC and NE signaling from the HPA axis and the LC/NE system. The neurophysiological core of this stress-learning integrative process is brain-wide BDNF-dependent GC/GR binding modulated by excitatory NE input released from the LC.

Thus, the CAN appears to be integral to learning, suggesting a need in learning theory to feature allostasis of the CAN and the socio-emotional stability associated with controlled levels of stress. In any event, the instrumental role of stress in children’s learning and the inseparability of stress responsivity from learning and memory forms a fundamental theoretical rationale for the CAN’s potential influence more specifically on reading and dyslexia.

Indeed, the second main outcome of this review suggests the centrality of the CAN to a general theory of reading acquisition. Like all learning, beginning reading instruction is accommodated by allostasis of the CAN, which reflects minimal stress exposure or successful adaptation to early and current stressful events. Such children are well-prepared for most learning expectations of formal schooling. However, an aspect of reading that makes it uniquely more difficult to teach and to learn is that learning to read requires reorganization of a left hemisphere extended brain circuit, centered on the VWFA, that may have been genetically selected by evolution as an adaptation for a different function: the integration of visual-spatial perceptual information. Successful beginning readers need to be receptive to repurposing this circuit for learning the print-sound associations of the alphabetic code. Regularization of the HPA axis facilitates that transition. Secondly, a key feature of the extended reading circuit is bottom-up LC/NE signaling to modulate the right hemisphere attention networks and their interhemispheric inhibitory controls, via the VOF, over letter–sound correspondence in the VWFA. Thus, both arms of the CAN provide key functions to support beginning reading. In effect, it is not an overstatement to conclude that the spread of literacy in society may depend on efforts to lessen exposure to severe and chronic stress during pre-natal, post-natal and early child development.

A third main outcome extends my previous reviews of dyslexia with the aim of formulating a more refined evolutionary model. The important focus of each previous paper is “built-in” as a valid assumption in the current review and treated more-or-less casually. Therefore, for a more detailed and comprehensive understanding of the model, the previous reviews should be consulted. Nonetheless, the current paper stands alone as complete and current. The earlier reviews of dyslexia featured: (1) brain networks and the epigenetic landscape (Kershner 2019a); (2) centrality of the right hemisphere (Kershner 2019b; (3) stress response of the HPA axis and LC/NE system as an evolutionary protective adaptation (Kershner 2020); (4) dyslexia as normal evolutionary variability (Kershner 2021a); and (5) Locus coeruleus interactions with the attention networks (Kershner 2021b).

The current review adds significant new dimensions to the model. One: the obligatory pairing and mutually reinforcing role in evolution and development, beginning in utero, of stress system allostasis and reading readiness. Although ELS may not invariably lead to dyslexia, there can be no doubt that overall mental and physical health and academic achievement including reading ability are at risk. Therefore, increased awareness of ELS alone can reduce the incidence of deleterious clinical and educational consequences. This calls for efforts to improve the quality of maternal care and children’s environment in early development. Classroom interventions to reduce anxiety, such as musical and exercise therapy may also be helpful. Moreover, although we need a better understanding of their effects, there is a potential for pharmacological interventions known to decrease the activity of both the HPA axis and the LC/NE system (Tsigos et al. 2020).

Number two: the review suggested that the vigilant and habituation stress-overload adaptations that have evolved to offset the adverse effects of exposure to ELS also pose a risk for under-achievement in reading and dyslexia. The review found preliminary evidence consistent with both strategies. It appears that the vigilant strategy may be associated with the “garden variety” poor reader: reading under-achievement resulting from HPA epigenetic change and hyperproduction of cortisol. Vigilance appears to be triggered by chronic and severe ELS, with potential for debilitating effects on physical health, emotional stability, and learning. In theory, the vigilant strategy, by promoting early adaptability, offers some protection from ELS but is a continuing liability for later systemic negative consequences of excessive GC production. Thus, the vigilant strategy is associated with domain-general reading disability as well as poor educational achievement in other areas. In addition, support was found for an association of habituation with BDNF and developmental dyslexia: a domain-specific form of reading disability. We assume that severity of stress exposure will be a main determinant of which strategy is enabled, with habituation keyed to marginal levels because dyslexia is not linked to general cognitive impairments or prior emotional disturbance. And finally, the different altered stress system origins (HPA vs LC/NE) suggest that neuro-hormonal testing for this difference in children with reading problems could provide a diagnostic test for dyslexia. At this early point in relevant research, such a possibility is highly speculative but would fulfil a major clinical and educational need.

Number three: stress-induced alterations of BDNF may buffer the HPA’s production of GCs but amplify release of NE by the LC to the attention networks. This is the first theoretical evidence of a disassociation of the two arms of the CAN as key to the ELS causal chain leading to dyslexia. The DAN’s resulting glutamatergic overstimulation and loss of neuroplasticity has the potential for impeding the fidelity of down-stream forward inhibitory control and inhibitory feedback to the reading circuit (i.e., right IPS > left IPS > VWFA), via the VOF which is the final pathway between the left IPS and the VWFA. Indeed, research with individuals with dyslexia reported degraded connectivity between the VWFA and bilateral regions of the DAN (van der Mark et al. 2011) and a recent study presented evidence for a causal role in dyslexia of anomalies in inhibitory connectivity between the VOF and the VWFA (Di Pietro et al. 2023). Thus, atypical changes in BDNF trafficking provoked by ELS may trigger a causal chain leading to dyslexia by the end-point disruption of the functions of the VWFA, impairing children’s ability to learn the print–sound associations fundamental to beginning reading.

Number four: the review underscores the importance in dyslexia of inhibitory interneurons in the VOF’s aberrant coupling between the IPS and VWFA. Six major classes of interneuron have been identified, differentiated by whether they target down-stream excitatory neuronal dendrite, soma, initial axon segment, or other interneurons where they become disinhibitory. Therefore, the complexities of understanding dyslexia as a disruption of interneuron connectivity between the IPS and VWFA presents an enormous challenge for basic research. For the present, we know that the balance between the opposing synaptic conductances of excitation and inhibition will be corrupted. And we can predict that this loss of proportionality will upset the rate of neural firing in response to excitatory inputs, preventing the timed integration of print/sound signaling in the VWFA. It is also important to recognize that the model’s causal pathway leading to dyslexia stands on its own independently of an evolutionary context, and if supported by future studies will call for reshaping traditional views of dyslexia.

Finally, the current model has implications for the co-morbidity of reading and mathematical disabilities. While a detailed analysis is beyond the scope of this review, the present model is consistent with the literature base suggesting that their domain-specific neural signatures are driven through different connectivity pathways (e.g., Das and Menon, 2022; Pinheiro-Chagas et al. 2020). In contrast to the current dyslexia model, mathematical disability involves bilateral inflow and outflow aberrations interconnecting the anterior intra-parietal sulcus (aIPS) and superior parietal lobe (SPL) with the hippocampus and inferior temporal cortex (ITC), a subdivision of the ventral occipital temporal cortex (VOTC). The dyslexia shortfall in interconnectivity appears to involve the left posterior intra-parietal sulcus (pIPS) (White et al. 2023), with the VOF clearly identified as the top-down pathway to the VWFA which is posterior to the number form area (NFA) on the fusiform gyrus. However, the review also shows that co-morbidity may result as a domain-general learning disability in reading and math when ELS engages the vigilant adaptation. Thus, it appears that dedicated domain-specific pathways are not involved in reading/math co-morbidity.

In conclusion, such distributed interconnectivity involving cortical/subcortical, rostral/caudal, and the corpus callosum (CC) engaging both cerebral hemispheres presents a comprehensive neurophysiological model of dyslexia. In theory, by dampening the ELS production of cortisol, habituation shelters against its long-term toxicity and promotes adaptive fitness. However, excessive release of NE into the attention networks, followed by disrupted coupling with the reading circuit, which is unprotected by natural selection, may place children at risk for dyslexia. In short, developmental dyslexia may be the collateral cost of a positive adaptation to ELS that disassociates the responsivity of the HPA axis from the LC/NE system. And finally, given the absence of directly supportive experimental evidence, this model should be viewed cautiously as a blueprint calling for longitudinal research to refine and directly test its theoretical parameters.

## Declarations

### Data Availability

No datasets were generated or analysed during the current study.
